# Three-Dimensional Printing Properties of Polysaccharide Hydrocolloids–Unrinsed Sturgeon Surimi Complex Hydrogels

**DOI:** 10.3390/foods11192947

**Published:** 2022-09-21

**Authors:** Kang Liu, Nana Zhao, Chenxi Xiang, Yujin Li, Xiaoming Jiang, Mingyong Zeng, He Xu, Haiyan Wang, Haohao Wu, Xiaoqing Yu, Yuanhui Zhao

**Affiliations:** 1 College of Food Science and Engineering, Ocean University of China, Qingdao 266003, China; 2 Qingdao Engineering Research Center for Preservation Technology of Marine Foods, Qingdao 266003, China; 3 Qingdao Institute for Nutrition and Health Innovation, Qingdao 266237, China; 4 Jiangsu Baoyuan Biotechnology Co., Ltd., Lianyungang 222100, China; 5 Hisense (Shandong) Refrigerator Co., Ltd., Qingdao 266100, China; 6 Marine Science Research Institute of Shandong Province, Qingdao 266104, China

**Keywords:** sturgeon, unwashed surimi, 3D printing, hydrogel, rheology

## Abstract

Herein, the microstructure and mechanical properties of hydrogels consisting of unrinsed sturgeon surimi (URSS) and plant-derived polysaccharides such as κ-carrageenan (KC), konjac gum (KG), xanthan gum (XG), guar gum (GG) and sodium alginate (SA), were studied by texture analysis, rheological measurement and scanning electron microscopy (SEM). Rheological results showed that the apparent viscosity, storage modulus (G′) and loss modulus (G″) of URSS increased by addition of KC, KG, GG and SA. The gel strength of resultant surimi products fabricated with KG/URSS mixture was significantly higher than that of other groups. KG could significantly improve the hardness (44.14 ± 1.14 N), chewiness (160.34 ± 8.33 mJ) and cohesiveness (0.56 ± 0.02) of the unrinsed surimi gel. Adding SA and KC had no significant effect on the textural characteristics of printed gels. However, an apparent decrease in the relevant mechanical properties of printed hydrogels was observed when XG and GG were added into surimi. SEM indicated that the incorporation of KG and KC could further integrate the gel structure of URSS as compared to hindering the cross-linking of surimi protein by XG and GG, which were in accordance with gel strength and water-holding capacity. These results provided useful information to regulate the 3D printing performance in functionalized surimi-based material.

## 1. Introduction

Three-dimensional printing technology is a bottom-up manufacturing method, achieving the printing requirements by accumulating materials layer by layer [1,2]. In the food field, 3D printing can provide targeted nutritional fortification and personalized customization according to the needs of different consumers, and it contributes to the manufacture of exquisite and complex food shapes that cannot be obtained by traditional processing methods [3]. The internal intricate structure could lead to new textural properties of final printed products [4]. Moreover, it can also broaden further the practical applications of available food materials [5]. In recent years, it has been reported that many food materials, such as chocolate [6], cheese [7], dough [8,9] and lemon juice gel [10], could be used for printing directly or by adjusting material properties such as rheological and mechanical properties.

High-quality surimi products are becoming increasingly popular among consumers because of their high nutrition and unique taste [11,12]. It is an indispensable green food for a high-quality life. Sturgeon (Acipenser) is referred to as a “living fossil” with high edible and economic value [13]. Its meat is rich in protein and essential amino acids and has no muscle spikes, which is very suitable for high-quality surimi processing [14]. Generally, traditional surimi products are prepared using frozen surimi [15]. Rinsing is a key step in the processing of traditional surimi. Pigment, inorganic ions, fat and water-soluble protein in the fish meat are removed by rinsing, thereby greatly improving the gel properties of surimi [16,17]. However, at the same time, multiple rinsings will also lead to the loss of nutrients and a decline in product yield, as well as a waste of water resources and environmental pollution [18]. As a viscous food gel system, surimi has been reported to be available for printing and has developed a variety of three-dimensional surimi with a strong appeal to consumers [19]. Wang et al. [19] proposed that the surimi gel prepared by adding 1.5% NaCl to silver carp surimi can be used as a food material suitable for three-dimensional printing. Dong et al. [20] showed that transglutaminase could effectively improve the 3D printability of Scomberomorus niphonius surimi, especially when the enzyme concentration was between 0.2% and 0.3%. However, different from washed surimi, unwashed surimi has higher fat content, resulting in poor gel-forming ability and viscoelasticity, which is not conducive to three-dimensional printing. Therefore, it is necessary to add additives to improve the rheological properties of unrinsed surimi to meet the requirement of 3D processing.

Hydrocolloids are often used as structural modifiers to change flow behavior (viscosity) and mechanical solid property of food [21,22]. Particularly interesting to form hydrogels are KC, KG, XG, GG and SA, which are considered the representative gel-forming polysaccharides, with thermoreversible gelling capacity [23]. Yang et al. [24] prepared PCS/SA composite membranes and evaluated their shape-memory properties, where the PCS with higher contents of SA possess better and faster shape recovery speed. The printability of taro paste was explored by adding different additives such as SA, carboxymethyl cellulose, whey protein, GG and XG [25]. KG and KC are commonly used in meat and seafood to provide the required texture, stability and water adhesion [26]. Moreover, the stability of printed products during postprocessing can also be improved by hydrocolloids [27]. So far, there is no research involving the effect of polysaccharide hydrocolloids on the 3D printing characteristics of surimi gel.

This study aimed to reveal the 3D printing properties of polysaccharide hydrocolloids–unrinsed sturgeon surimi complex hydrogels. First, the five polysaccharide hydrocolloids–unrinsed sturgeon surimi complex hydrogels were prepared. Then, their apparent viscosity, viscoelastic properties, morphology features and microstructure were investigated. Finally, the results of texture profile analysis and water-holding capacity of 3D-printed surimi products were obtained.

## 2. Materials and Methods

### 2.1. Materials

Fresh hybrid sturgeon (Acipenser baeri Brandt ♀ × Acipenser schrenckii Brandt ♂) were obtained from an aquatic product company located in Chengyang District, Qingdao (Shandong, China), and transported immediately to the laboratory in a fresh state. The food-grade κ-carrageenan (KC), konjac gum (KG), xanthan gum (XG), guar gum (GG) and sodium alginate (SA) were purchased from Henan Wanbang Industrial Co., Ltd. (Zhengzhou, China).

### 2.2. Preparation of Surimi

The fresh hybrid sturgeon were knocked unconscious with force and then quickly stripped of their heads, viscera and skin. The obtained fish meat was cut into small pieces and minced by meat grinder (Model UM5, Stephan Machinery Co., Hameln, Germany). After that, 0.25% sodium tripolyphosphate and 4% sorbitol were used as the cryoprotectant and mixed directly with the unwashed surimi. Finally, the unwashed sturgeon surimi was packed into sealed bags (500 g per bag) and frozen at −18 °C for use within two weeks.

### 2.3. Preparation of Surimi Gels

Gluconolactone could induce the formation of unrinsed sturgeon surimi gel, and hydrocolloids could improve the rheological characteristics of polysaccharide hydrocolloids–unrinsed sturgeon surimi complex hydrogels based on 3D printing characteristics. To prepare surimi gel, the frozen unwashed surimi was taken out from the refrigerator and thawed at 4 °C overnight. The surimi was put in a chopper machine (National Model SY-5, Guangzhou, China) and chopped for 2 min, then salt (1.5%, *w*/*w*), gluconolactone (1%, *w*/*w*) and different hydrocolloids (0.5%, *w*/*w*) were added and chopped for another 2 min. Unrinsed surimi gel without hydrocolloid was used as the control group. The moisture content in surimi was around 80%. Finally, the chopped surimi was placed at 4 °C for 24 h to form a hydrogel.

### 2.4. Rheological Properties

The apparent viscosity of the surimi was measured with a rheometer (MCR-101, Anton-Par, Germany) with the shear rate range of 0.01–10 s^−1^. The raw surimi slurry was placed between the rheometer platform and a parallel plate (20 mm in diameter) with a gap of 1 mm. It was allowed to relax on the rheometer platform at 25 °C for 1 min before starting the test to reach a stable state.

Preliminary experiments of strain sweeping were carried out at a constant frequency of 1 Hz in the strain range of 0.01–500% to determine the linear viscoelastic region. Within the identified linear viscoelastic region (at 1% strain), the viscoelastic characteristics of surimi were evaluated in the frequency range of 0.1 to 10 Hz. All the experiments were repeated three times.

### 2.5. Three-Dimensional Printing Process

**Figure 1:**
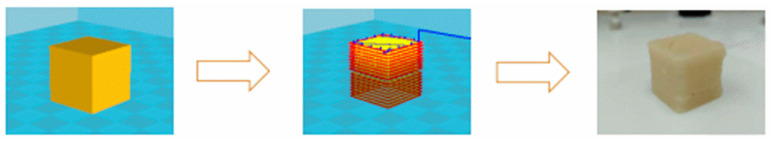
The general process of 3D printing.

The 3D printing process was carried out using a piston extrusion-based three-dimensional printer (SHINNOVE, Shiyin Technology Co., Ltd., Hangzhou, China). The printed model was designed on the computer and then cut into a monolayer outline by the open-source software of CURA 15.02.1 (Ultimaker B.V., Utrecht, The Netherlands). Then, the printer printed layer-by-layer according to the designed printing path, and finally formed a three-dimensional pattern by superimposing the layers (Figure 1). The printing parameters in this experiment were set as follows: the maximum volume of the syringe was 30 mL, the nozzle diameter was 1.20 mm, the printing speed was 20 mm/s, the filling density was 100%, and the whole printing process was carried out at room temperature (around 25 °C).

In order to evaluate the printing quality, the printing behavior was closely observed and recorded. The photos were taken immediately after printing, and the size of the printed surimi was measured with a vernier caliper. The printed cylindrical sample (25 mm diameter and 20 mm height) was heated in a water bath at 90 °C for 30 min, then immediately cooled with crushed ice. All samples are stored at 4 °C for the determination of the following indicators.

### 2.6. Scanning Electron Microscopy (SEM)

To observe the internal microstructure of the surimi, the raw surimi and the cooked printed surimi were cut into 1 mm-thick slices and freeze-dried. Then, the dried samples were gold-coated and observed under a SEM (JSM-5800 LV, JEOL, Tokyo, Japan) at an accelerating voltage of 20 kV.

### 2.7. Gel Properties of 3D-Printed Surimi

#### 2.7.1. Texture Analysis

The printed cylindrical samples (25 mm diameter and 20 mm height) were cooked, then immediately cooled with crushed ice. The gel strength was measured using a texture analyzer (TMS-Pro, Food Technology Co., Sterling, VA, USA) equipped with a spherical plunger of 5 mm diameter at room temperature. The probe was used for puncture test at a constant moving speed of 1 mm/s and the puncture distance was 10 mm [28].

The texture was determined based the method by Chéret et al. [29]. The texture characteristics of surimi cylinder were analyzed by the texture analyzer with a plate of 35 mm diameter, including hardness, springiness, chewiness, adhesiveness, cohesiveness and gumminess. The final result was obtained by compressing the surimi samples twice at a speed of 1 mm/s and a deformation of 40%.

#### 2.7.2. Water-Holding Capacity (WHC)

The water-holding capacity was determined based the method by Vega-Warner et al. [30]. The surimi gel was first weighed (marked as W_1_), then wrapped in double-layer filter paper and centrifuged at 6860 *g* for 10 min. After centrifugation, the sample was weighed again (marked as W_2_). The value of WHC was calculated using Equation (1).

WHC (%) = [1 − (W_1_ − W_2_)/W_1_] × 100 (1)

### 2.8. Statistical Analysis

One-way analysis of variance (ANOVA) was performed using the software of SPSS statistics 24 (IBM, Armonk, NY, USA), and mean comparison was tested by Duncan multirange test and the significance of the data was analyzed (*p* < 0.05). The data were graphed and analyzed by Origin 2017.

## 3. Results and Discussion

### 3.1. Effect of Polysaccharides on the Rheological Properties of Unrinsed Surimi Gel

The mechanical and rheological properties of materials based on extrusion printing has a relatively large impact on the ability of samples after printing to maintain the shape and the supporting force between layers [31]. The ideal paste should exhibit a certain viscous behavior to ensure that it can be smoothly squeezed out through the nozzle and require sufficient mechanical strength to support its 3D printing structure [32,33]. In this experiment, the viscosity and viscoelasticity of surimi were analyzed to understand the relationship between rheology and 3D printability.

#### 3.1.1. Apparent Viscosity

**Figure 2:**
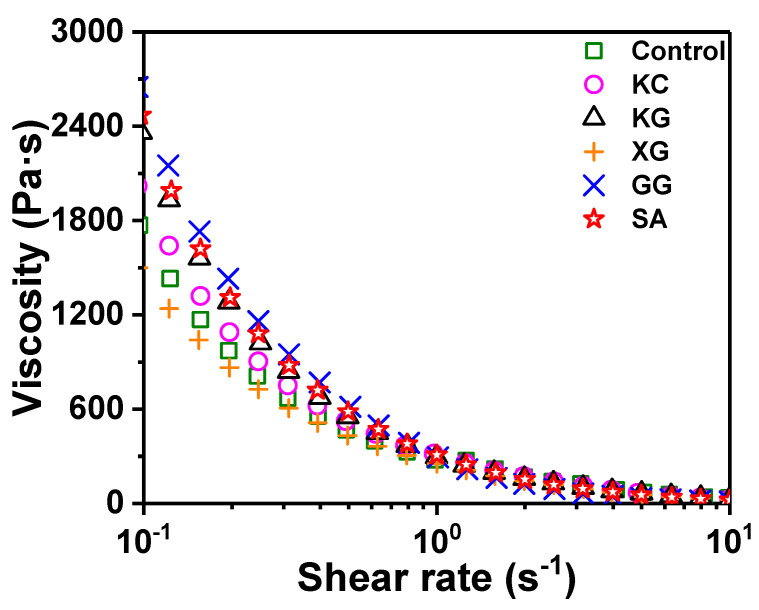
Effects of different polysaccharides on apparent viscosity of unwashed surimi gel. KC: κ-carrageenan; KG: konjac gum; XG: xanthan gum; GG: guar gum; SA: sodium alginate.

The viscosity of the material is an important indicator of whether it can be extruded smoothly from the nozzle [33,34]. The low viscosity exhibited at high shear rate is conducive to the extrusion of the material, and the high viscosity exhibited at low shear rate helps to support its own structure [35,36]. The curve of the apparent viscosity of the surimi material changing with the shear rate is presented in Figure 2. The viscosity gradually decreased with the increase in shear rate, indicating that the surimi gel has shear-thinning properties. After adding different polysaccharides, except XG, the apparent viscosity increased to varying degrees. The highest apparent viscosity was obtained for surimi containing GG, followed by SA. Ramírez et al. [37] believed that some polysaccharides could be embedded in a continuous matrix formed by water and proteins in surimi and filled with gel to improve the mechanical and functional properties of surimi and restructured fish gels.

#### 3.1.2. Viscoelastic Properties

**Figure 3:**
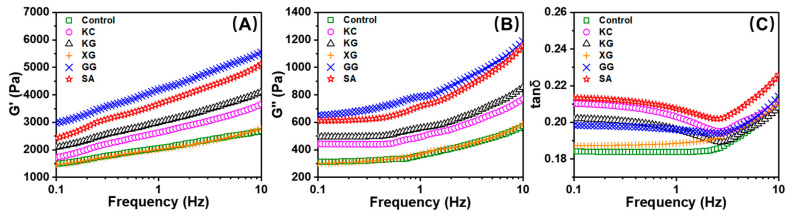
Effect of different polysaccharides on the dynamic rheological characteristics of unwashed surimi gel. (**A**) Storage modulus (G′); (**B**) loss modulus (G″); (**C**) loss tangent (tanδ). KC: κ-carrageenan; KG: konjac gum; XG: xanthan gum; GG: guar gum; SA: sodium alginate.

G′ and G″ represent elastic solid-like behavior and viscous behavior, respectively. Higher G′ can reflect strong mechanical strength, which helps to display excellent self-supporting ability after deposition [33]. Figure 3A,B display the G′ and G″ of surimi with different polysaccharides during frequency scanning. As can be observed, the increase in G′ and G″ depends on frequency, which is characteristic of weak gel. Moreover, the G′ of all samples is always higher than G″ in the linear viscoelastic region, showing solid-like properties. The addition of KC, KG, SA and GG increases the G′ of the surimi, while XG decreases it. This indicates that XG weakens the gel structure of unrinsed surimi. Tanδ is the ratio of G″ to G′, used to characterize viscoelastic properties [33]. The tanδ value is lower than 1, indicating that the material is biased towards elastic characteristics, while the material with tanδ value above 1 performs more viscous characteristics with good liquidity [38]. The addition of polysaccharides increases the fluidity of surimi (Figure 3C). In the frequency range of 0.1~10 Hz, the highest tanδ value was obtained for the unrinsed sturgeon surimi gel containing SA. The unrinsed surimi gel without polysaccharide had the lowest tanδ value, suggesting that its rheological properties were more solid-like and had poor fluidity. This could explain the severe line breakage of the control sample during printing.

### 3.2. Evaluation of Printing Effect and Size Deviation

**Figure 4:**
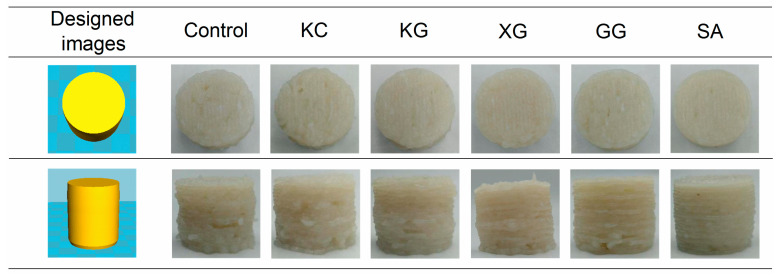
Three-dimensional printing effect of unwashed surimi with different polysaccharides. KC: κ-carrageenan; KG: konjac gum; XG: xanthan gum; GG: guar gum; SA: sodium alginate.

Since surimi gel has a shear-thinning behavior, its low viscosity makes it easy to be squeeze out of the nozzle [19]. However, the addition of different polysaccharides has significantly different effects on the printing process and printing effect. Figure 4 shows the printed model of the solid cylinder (diameter × height = 25.0 mm × 20.0 mm) and the photos of surimi material after printing. The designed model is relatively simple but there are many layers, so different degrees of difference between layers and layer structure can be seen in printing. From the printing process and photos after printing, obvious traces of broken strips could be observed in the printed control samples, which led to printing structure chaos, and even produced deformation as the printing continued (Figure 4). For samples containing KC and KG, respectively, the printing ability improved, but the appearance of the printed surimi was rough. This is due to the lower G′ and viscosity. XG improved extrusion fluency but showed poor self-supporting performance. Kim et al. [27] found that the addition of polysaccharides resulted in an increase in G′ of the dough, and high G′ was beneficial to supporting the sediment layer and maintaining structural stability. The addition of guar gum and sodium alginate significantly improved the mechanical strength of the material and maintained the printing structure well. However, it could be seen from the picture of GG that the extruded lines were not continuous in the early stage of printing. This is because the high viscosity of GG made it difficult to squeeze out. In the unwashed surimi with SA, there were few interruptions during the whole printing process. After printing, the structure between layers of surimi was tight and the overall appearance was smooth. The printing structure was complete, which was the closest to the printing model.

In order to further reflect the precision of the printed products, the actual height and diameter were measured (Table 1). Compared with the control sample, the surimi gel with polysaccharides was closer to the design value in height and diameter, which improved the printing accuracy. The sedimentary layer structure of the control sample was not close, so the height of the sample deviated from the model value, while the sodium alginate group had good self-supporting ability and fluidity due to the appropriate elastic modulus and viscosity, and piled up closely from layer to layer. The height deviation and diameter deviation (absolute value) were the smallest, only 2.80% and −0.40%, respectively.

### 3.3. Scanning Electron Microscopy (SEM)

**Figure 5:**
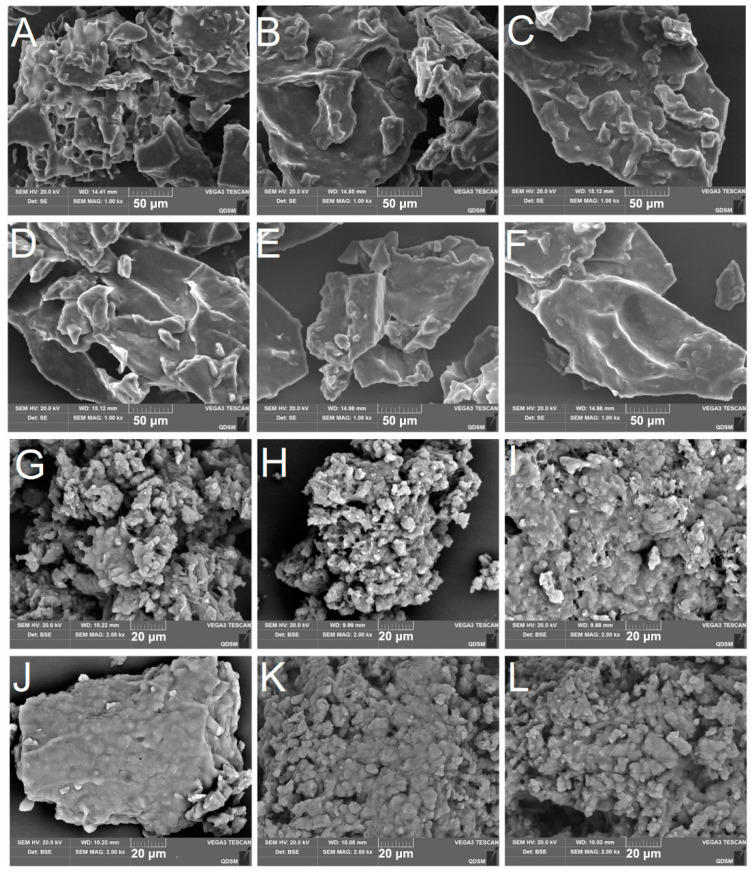
Electron microscope pictures of raw surimi paste and cooked surimi with different polysaccharides. Raw surimi paste: (**A**–**F**) at magnification 1000×, cooked surimi: (**G**–**L**) at magnification 2000×. AG: control; BH: κ-carrageenan (KC); CI: konjac gum (KG); DJ: xanthan gum (XG); EK: guar gum (GG); FL: sodium alginate (SA).

The microstructures of the raw surimi paste and the 3D-printed surimi after cooking are shown in Figure 5. For raw surimi materials, the surface of the control was very rough and fractured, with obvious holes, which was the reason for the fracture and discontinuity of the print lines mentioned above. KC and KG also showed similar characteristics to the control, consistent with the printing effect. In comparison, a more continuous structure could be observed in the photos of GG and XG, with no holes on the surface but still somewhat rough. The surface of SA was smooth, showing a continuous and tight structure without fracture, which also exhibited better effect and printing accuracy in the printing process. This may be due to the good compatibility of SA with the network structure of the unrinsed surimi gel, so the structure of the surimi gel is improved and shows good rheological properties.

From the microstructure of the cooked surimi samples, it could be seen that the surimi gel network structure without polysaccharides was porous and irregular. After the addition of polysaccharides, the microstructure of the surimi became smooth and the pores were reduced or even invisible, indicating that polysaccharides are filled in the surimi protein and improved the gel network structure. However, the cross-linking of surimi proteins could be blocked by XG and GG, causing the proteins to condense into colloidal blocks. The effect of XG was more significant. The adverse effects of XG on the structure of surimi gel may be related to the mutual repulsion of its anionic properties and the anionic charge of myofibrillar protein [39]. SA filling in the surimi protein network structure makes the surface of the microstructure of surimi look more continuous and smooth, but in fact has little effect on the gel structure.

### 3.4. Effect of Different Polysaccharides on Gel Properties of 3D-Printed Surimi Products

#### 3.4.1. Texture Analysis

**Figure 6:**
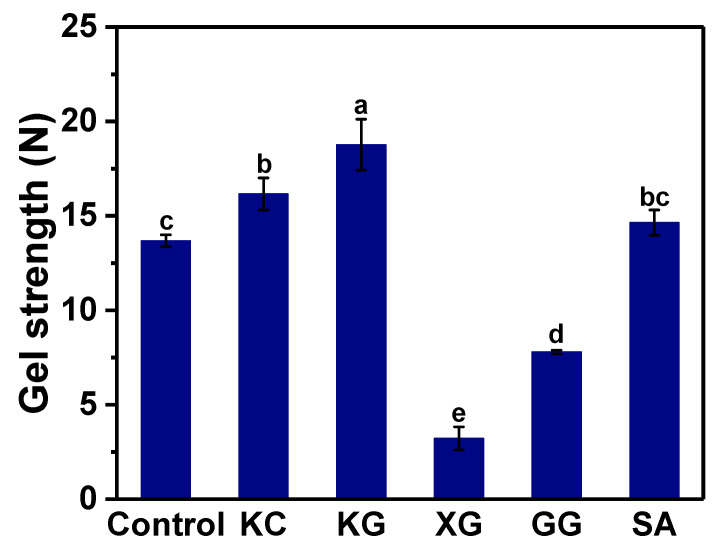
Gel strength of printed cylindrical surimi with different polysaccharides after cooking. KC: κ-carrageenan; KG: konjac gum; XG: xanthan gum; GG: guar gum; SA: sodium alginate. Bars indicate the standard deviation (n = 5). Lowercase letters on the bars indicate significant differences (*p* < 0.05).

Gel strength is one of the important indexes to measure the quality of surimi products, which can reflect the firmness of the gel network structure. The effect of adding different polysaccharides on the gel strength of cooked surimi is shown in Figure 6. The results show that compared with the control, except for XG and GG, the other three polysaccharides improved the gel strength of surimi to varying degrees. The addition of different polysaccharides has different effects on the characteristics of surimi gel. KC is anion polysaccharide, which can form a gel during the cooling process and enhance the original protein network structure [40]. KG can form a thermal irreversible gel [41]. Therefore, during the heating process, konjac gel can further strengthen the network structure formed by the deformation, extension and cross-linking of fish myosin molecules, and improve the gel strength. However, as nongelling polysaccharides, XG and GG cannot interact with surimi protein and hinder the formation of the surimi protein network structure, thereby reducing the gel strength. SA enhanced the gel strength of surimi, but not significantly. They found that both KC and KG could improve the gel strength during the formation of myosin gel, and the addition of konjac gum to mincemeat cod could increase the gel strength by 8~10 times [42,43]. Verbeken et al. [44] found that carrageenan can increase the strength of myofibril protein heat-induced gel.

Texture profile analysis (TPA) of surimi was obtained by using a texture analyzer to simulate the secondary chewing of food in the human mouth, which was the internal physical property of the sample [45]. As shown in Table 2, the TPA parameters of surimi were significantly affected by the addition of polysaccharides (*p* < 0.05). Compared with the control group, KG significantly improved the hardness, chewiness and cohesiveness of the unrinsed surimi gel, but the increase in springiness, adhesiveness and gumminess was not obvious. This is because the gel structure formed by the KG itself during the heating process strengthens the network structure of myosin molecules and makes the internal structure more stable. The texture properties of KC, SA and the control were not significantly different, while XG and GG significantly reduced the texture properties, among which xanthan gum decreased the most (*p* < 0.05). This indicates that both XG and GG are not conducive to gel formation. Pérez-Mateos et al. [46] demonstrated that adding GG may destroy the network structure of surimi gel, thereby reducing the hardness of blue whiting surimi gel. KG can significantly increase the gel strength of cod surimi [42]. These results are similar to this experiment.

#### 3.4.2. Water-Holding Capacity (WHC)

**Figure 7:**
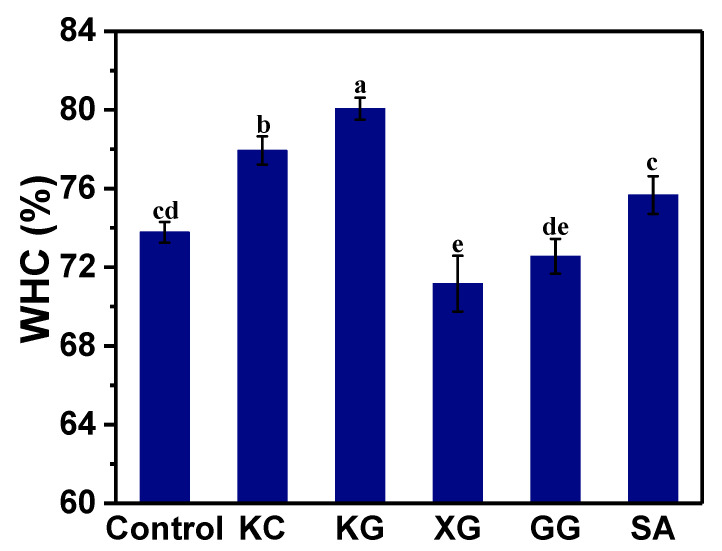
Water-holding capacity (WHC) of 3D-printed unwashed surimi gel after cooking. KC: κ-carrageenan; KG: konjac gum; XG: xanthan gum; GG: guar gum; SA: sodium alginate. Bars indicate the standard deviation (n = 5). Lowercase letters on the bars indicate significant differences (*p* < 0.05).

Figure 7 shows the influence of different polysaccharides on the water retention of 3D-printed surimi. The water holding capacity of surimi gel is related to the protein–water interaction in the gel network [47]. The more water molecules held in the protein network matrix, the stronger the water retention. As shown in Figure 6, the WHC of KG and KC was significantly higher than that of other groups (*p* < 0.05). KG had the largest water-retention capacity, while the WHC of XG was only 71.17%, which was the weakest. Compared with the control group, SA and GG had an effect, but not significantly. In thermally induced gelation, the gel network is formed when the water is combined or other components are trapped. The addition of KG and KC resulted in a significant increase in WHC, which was due to the strengthening of the protein network structure to bind more water and water-soluble substances (*p* < 0.05). This is consistent with the previous gel strength results. Park [48] found that certain polysaccharides added to surimi products can form gels and interweave with the network structure of surimi protein, thereby increasing the strength of surimi gel. At the same time, the polysaccharides absorb water and swell as the temperature rises, trapping water molecules in the network structure and enhancing the water-holding capacity of the gel [48]. The adverse effects of XG on surimi gel may be related to the mutual repulsion of its anionic properties and the anionic charge of myofibrillar protein [39].

## 4. Conclusions

The addition of KC, KG, GG and SA obviously increased the apparent viscosity and mechanical strength (G′), while XG decreased the apparent viscosity of unwashed surimi. KC and KG improved printing performance, but the printed objects showed rough surface structure and large deviation from the target model. The printed objects using surimi with XG exhibited poor shape-retention ability due to the lower G′. The addition of GG provided good self-supporting properties and printability for unrinsed surimi, but the excessively high viscosity made the extrusion discontinuous in the early stage of printing. The unrinsed surimi gel with SA showed the best printing quality and highest printing precision. Furthermore, SA had no significant effect on the physical properties of cooked surimi. In short, SA can effectively improve the printing characteristics of the unrinsed sturgeon surimi gel and is expected to develop unwashed surimi products with high printing quality.

## Figures and Tables

**Table 1:** Printing time, weight and dimension deviation of raw surimi ink.

Group	Printing Time (min)	Weight (g)	Height (mm)	Height Deviation (%)	Diameter (mm)	Diameter Deviation (%)
Control	13.63 ± 0.01 ^a^	10.14 ± 0.02 ^a^	20.92 ± 0.04 ^e^	4.60 ± 0.20 ^e^	24.47 ± 0.01 ^a^	−2.13 ± 0.05 ^a^
KC	13.62 ± 0.01 ^a^	10.16 ± 0.04 ^a^	20.75 ± 0.05 ^c^	3.75 ± 0.25 ^c^	24.65 ± 0.02 ^c^	−2.17 ± 0.58 ^a^
KG	13.61 ± 0.01 ^a^	10.19 ± 0.04 ^a^	20.75 ± 0.03 ^c^	3.77 ± 0.14 ^c^	24.55 ± 0.02 ^b^	−1.80 ± 0.09 ^a^
XG	13.61 ± 0.02 ^a^	10.18 ± 0.03 ^a^	20.84 ± 0.02 ^d^	4.20 ± 0.12 ^d^	24.77 ± 0.02 ^d^	−0.92 ± 0.07 ^bc^
GG	13.60 ± 0.02 ^a^	10.13 ± 0.04 ^a^	20.65 ± 0.04 ^b^	3.25 ± 0.20 ^b^	24.76 ± 0.02 ^d^	−0.97 ± 0.08 ^b^
SA	13.59 ± 0.03 ^a^	10.18 ± 0.03 ^a^	20.56 ± 0.03 ^a^	2.80 ± 0.14 ^a^	24.90 ± 0.02 ^e^	−0.40 ± 0.07 ^c^

Data are means ± SD (n = 5). Different lowercase letters in the same column indicate the significant differences among samples (*p* < 0.05). KC: κ-carrageenan; KG: konjac gum; XG: xanthan gum; GG: guar gum; SA: sodium alginate.

**Table 2:** Texture profile analysis (TPA) indicators for 3D-printed cylindrical surimi after cooking.

Sample	Hardness (N)	Springiness (mm)	Chewiness (mJ)	Cohesiveness	Adhesiveness (mJ)	Gumminess (N)
Control	35.48 ± 2.54 ^b^	6.40 ± 0.13 ^a^	120.09 ± 6.63 ^b^	0.50 ± 0.01 ^b^	0.04 ± 0.01 ^c^	20.51 ± 2.97 ^ab^
KC	40.21 ± 4.54 ^ab^	6.33 ± 0.04 ^a^	135.20 ± 22.25 ^b^	0.50 ± 0.03 ^b^	0.05 ± 0.01 ^c^	21.34 ± 3.43 ^ab^
KG	44.14 ± 1.14 ^a^	6.47 ± 0.06 ^a^	160.34 ± 8.33 ^a^	0.56 ± 0.02 ^a^	0.04 ± 0.01 ^c^	24.78 ± 1.06 ^a^
XG	9.17 ± 1.95 ^d^	4.30 ± 0.10 ^c^	9.62 ± 0.25 ^d^	0.24 ± 0.01 ^d^	0.11 ± 0.01 ^a^	2.43 ± 0.25 ^c^
GG	21.67 ± 1.14 ^c^	5.95 ± 0.10 ^b^	39.27 ± 4.15 ^c^	0.30 ± 0.02 ^c^	0.07 ± 0.01 ^b^	6.59 ± 0.62 ^c^
SA	37.44 ± 2.87 ^b^	6.44 ± 0.11 ^a^	128.43 ± 9.04 ^b^	0.53 ± 0.01 ^ab^	0.04 ± 0.01 ^c^	19.89 ± 1.82 ^b^

Data are means ± SD (n = 5). Different lowercase letters in the same column indicate the significant differences among samples (*p* < 0.05). KC: κ-carrageenan; KG: konjac gum; XG: xanthan gum; GG: guar gum; SA: sodium alginate.

## Data Availability

Data are contained within the article.
